# The Cost of Ankylosing Spondylitis in the UK Using Linked Routine and Patient-Reported Survey Data

**DOI:** 10.1371/journal.pone.0126105

**Published:** 2015-07-17

**Authors:** Roxanne Cooksey, Muhammad J. Husain, Sinead Brophy, Helen Davies, Muhammad A. Rahman, Mark D. Atkinson, Ceri J. Phillips, Stefan Siebert

**Affiliations:** 1 College of Medicine, Swansea University, Swansea, Wales, United Kingdom; 2 Keele Management School, Keele University, Keele, Newcastle, England, United Kingdom; 3 College of Human and Health Sciences, Swansea University, Swansea, Wales, United Kingdom; 4 Institute of Infection, Immunity and Inflammation, University of Glasgow, Glasgow, Scotland, United Kingdom; Queen Mary University of London, UNITED KINGDOM

## Abstract

**Background:**

Ankylosing spondylitis (AS) is a chronic inflammatory arthritis which typically begins in early adulthood and impacts on healthcare resource utilisation and the ability to work. Previous studies examining the cost of AS have relied on patient-reported questionnaires based on recall. This study uses a combination of patient-reported and linked-routine data to examine the cost of AS in Wales, UK.

**Methods:**

Participants in an existing AS cohort study (n = 570) completed questionnaires regarding work status, out-of-pocket expenses, visits to health professionals and disease severity. Participants gave consent for their data to be linked to routine primary and secondary care clinical datasets. Health resource costs were calculated using a bottom-up micro-costing approach. Human capital costs methods were used to estimate work productivity loss costs, particularly relating to work and early retirement. Regression analyses were used to account for age, gender, disease activity.

**Results:**

The total cost of AS in the UK is estimated at £19016 per patient per year, calculated to include GP attendance, administration costs and hospital costs derived from routine data records, plus patient-reported non-NHS costs, out-of-pocket AS-related expenses, early retirement, absenteeism, presenteeism and unpaid assistance costs. The majority of the cost (>80%) was as a result of work-related costs.

**Conclusion:**

The major cost of AS is as a result of loss of working hours, early retirement and unpaid carer’s time. Therefore, much of AS costs are hidden and not easy to quantify. Functional impairment is the main factor associated with increased cost of AS. Interventions which keep people in work to retirement age and reduce functional impairment would have the greatest impact on reducing costs of AS. The combination of patient-reported and linked routine data significantly enhanced the health economic analysis and this methodology that can be applied to other chronic conditions.

## Introduction

Ankylosing Spondylitis (AS) is a chronic inflammatory arthritis characterised by spinal involvement, with pain, stiffness and reduced range of movement. AS typically starts in the second or third decades of life, and affects patients’ functioning and quality of life, with a significant burden in terms of work impairment, early retirement, lifetime health and social care resource utilisation [1–18].

A study investigating AS-related healthcare costs in three different European countries, estimated costs at £2253/patient/year [17]. This study used patient-reported visits to healthcare professionals to calculate costs. Separately the authors also examined work status and productivity costs in the same cohort and noted significant differences between countries. Costs ranged between £406 and £1073 using the frictional cost method (employers’ perspective) and £3080–£7561 using the human capital approach (patients’ perspective). These large differences between countries makes it difficult to generalise work-related costs beyond the country of origin and suggests the majority of costs may be difficult to quantify. A study in the United Kingdom explored healthcare and productivity losses using patient-reported outcomes and the human capital approach and found that on average, AS total costs over a period of three months were £2802 (or £11208/person/year)(1). Healthcare-related costs contributed a small proportion of the costs (just 15%) while work-related costs (unemployment, absenteeism and reduced work productivity) accounted for the majority of AS-related costs. However, as with previous studies these estimates are based entirely on patient-recall of healthcare visits, which have well recognised limitations [19].

We have previously suggested that linked routinely collected healthcare data allows the entire patient journey to be mapped more accurately, both retrospectively and prospectively, thereby enhancing the data available for health economic analysis [20]. In this current study we use linked patient-reported data and routinely collected medical record data to estimate the total cost of AS in the UK, including healthcare resource utilisation, work productivity loss and out-of pocket AS-related costs. Therefore, this paper and the novel methodology utilised makes a significant contribution to the cost of illness literature in general as well as our understanding of the true costs of AS.

## Materials and Methods

### Ethics Statement

The study and amendments had full ethical approval from the London Multi-centre Research Ethics Committee and was run in accord with Good Clinical Practice and the Declaration of Helsinki. All participants gave written consent, including consent for data linkage.

### Participants and Healthcare System

Participants were part of a previously established population-based AS cohort created as part of an Medical Research Council (MRC)/National Institute for Social Care and Health Research (NISCHR) funded Patient Research Cohort Initiative, previously described [21]. The cohort recruited 570 people with a diagnosis of AS confirmed by a rheumatologist, from across Wales in the UK. Wales has the National Health Service (NHS) a universal healthcare system that provides free medical care and is funded through taxes.

### Patient-Reported Data

Participants were invited to complete questionnaires and return by post or online between mid-2009 and mid-2010, which collected demographics, disease severity, work and activity limitations, out-of-pocket expenses, transport to healthcare appointments and carer assistance. Individuals were contacted up to a total of two times following initial contact before considered as a non-responder.

### Work Limitation Questionnaire (WLQ)

The WLQ explores the degree of limitations experienced due to chronic health problems and while work has not yet validated the WLQ is AS, the validity and reliability of the instrument has been demonstrated in osteoarthritis [22]. Comprising 25 items, the WLQ invites respondents to rate their level of difficulty or ability to perform work-specific demands. The items of the WLQ are grouped into four scales; Time management, physical demands, mental-interpersonal and output demands scales. The scale scores range from 0 (limited none of the time) to 100 (limited all of the time). An algorithm converts the WLQ scale scores into an estimate of productivity loss as a result of AS [23].

### The Work Productivity and Activity Impairment (WPAI)

The WPAI Questionnaire is a quantitative measure that yields scores on absenteeism, presenteeism, work productivity loss and activity impairment that has been validated in AS [24]. The six-item WPAI investigates whether in current employment, the hours missed at worked due to AS and other reasons, hours work and the degree to which AS affects productivity while working or doing regular activities. Impairment scores are calculated and expressed as impairment percentages, with higher numbers indicating greater impairment and less productivity [25].

### Patient-Reported Healthcare Resource Use

Participants were asked to recall various AS-related events over the preceding 3 month period, (visits to health professionals, transport, investigations performed, medication; prescribed and over-the-counter (OTC), adaptations to the home/car, carer assistance, self-funded visits to health professionals (e.g. private physiotherapy).

### Measures of Disease Severity and Quality of Life

Disease-specific measures included the validated and routinely-used Bath Ankylosing Spondylitis Disease Activity Index (BASDAI) [26] and Bath Ankylosing Spondylitis Functional Index (BASFI) [27]. Quality of life was assessed using the EuroQoL EQ-5D [28] and EQ-VAS for Health Status from 0 (worst imaginable health state) to 100 (best imaginable health state).

### Linked Routine Data

The Farr institute of Health Informatics Research comprises four nodes distributed across the UK. One of the nodes, CIPHer (Centre for Improvement in Population Health through E-records), brings together routine health data using the Secure Anonymised Information Linkage (SAIL) databank [29, 30], which anonymously links a wide range of person-based data [31]. The range of complementary datasets include General Practice (GP) records, outpatient (OP), inpatient (IP) and accident and emergency (A&E) department data containing data regarding healthcare visits including reason for visit, medication administered and medical and surgical procedures. Complete hospital data for Wales is available in the SAIL databank and presently, 195 GP practices out of 499 contribute to the SAIL databank, yielding a39% coverage for Wales.

### Public Health Resource Use

Healthcare costs were calculated using a bottom-up micro-costing approach, which estimates the average cost of treatment patient/year using the unit costs combined with quantity of use. The costs are reported at 2010 current prices, in terms of average cost patient/year and 95% confidence intervals. The unit costs for healthcare use were obtained from a number of sources, for instance, unit costs related to GP activities are taken from Unit Costs of Health and Social Care by the Personal Social Services Research Unit (PSSRU) [32], where costs for a GP consultation lasting 17.2 minutes and 11.7 minutes cost £53 and £36, respectively. GP visits were assessed using patient-reported GP visits (questionnaires) and, where available, routine data held in GP Read codes [33]. GP Read code data, does not necessarily indicate a personal consultation or visit to the GP but may represent test results, letters, inputting data obtained from other healthcare settings etc. Exploration of read codes indicated that whenever two or more types of events were recorded in the codes on the same day (i.e. procedures, diagnosis or drugs) this was likely to represent GP visit and counted as such, while a single event on a particular day was not counted as a visit. Costs were attributed to that named GP event using the unit costs for administration (S1 Table: Unit Costs). Patient-reported and routine data GP visits were calculated separately and stratified by disease severity, function and age.

For prescription costs, the PSSRU 2010 reports average prescription cost issued from the GP surgery, however in our cost calculation we have used a weighted average (based on usage) of self-reported drugs prescribed for AS (painkillers, non-steroidal anti-inflammatory drugs (NSAIDS), disease modifying anti-rheumatic drugs (DMARDs), and Anti-TNF agents) obtained from the Prescription Cost Analysis (PCA) data for Wales 2010. We obtained a unit cost of £60.60 for the AS-related drugs. Unit costs for outpatient attendances are taken from NHS reference cost database (£137/attendance) which also costs diagnostic procedures such as investigations, tests, drugs, devices as well as other costs that do not generate a separate healthcare resource group. Diagnostic imaging including plain x-rays, MRI and other scans were calculated individually but are not reported separately due to their high volume and low cost. Therapy services were also included where referral for treatment has been made by a clinician, health professional or self-referral where the patient attends a discrete therapy clinic solely for the purpose of receiving therapy treatment (S1 Table: Unit Costs).

### Out-of-Pocket AS-Related Costs

The unit costs for transport used by the patients to attend healthcare facilities were obtained from various web sources (e.g. AA Motoring cost 2010, UK railway travel cost compiled by the Guardian [34]). Costs for OTC medications were calculated from the “prescription of Wales-2010” dataset [35]. Patients’ out-of-pocket expenses also included: (i) ‘adjustment’ costs for making changes to their home or car, (ii) costs incurred for appliances and aids (iii) exercise-related costs for AS and, (iv) one-off purchases AS-related purchases (S1 Table: Unit Costs).

### Work Productivity Loss

Work-related costs were estimated using the human capital approach [36, 37]. The cost components included early retirement, absenteeism (due to AS), presenteeism (due to AS) and unpaid work by carers (including visits to healthcare facilities). Age and sex-specific average daily wage rates obtained from the Office of National Statistics were used to value lost hours due to AS (S2 Table: Hourly pay rate, gross (£) for all employee jobs in the United Kingdom at 2010 prices).

### Statistical Analysis

The socio-demographics and clinical characteristics are presented using descriptive statistics accompanied by the 95% confidence intervals and bootstrapped confidence intervals (1000 iterations), where applicable. Regression analysis was used to account for age, gender, disease activity, functional impairment, quality of life and self-rated health status as correlates of cost. All analyses were performed using STATA 12.

### Factors Associated with Healthcare Costs

Patient-reported data and routine data were analysed separately. Comparison of routine with patient-reported data were used to derive yearly estimates of healthcare use. Prescription estimates used numbers of AS-related drugs. The average number of prescriptions is reported for the 3 months recall period within the questionnaire and is also available in the routine data. From routine data, 1 year and 5 year retrospective periods as well as 6 months prospectively from the questionnaire completion date were collected and stratified by disease severity and age.

### Factors Associated with Work Productivity Loss

Multi-predictor maximum-likelihood logistic regression and Ordinary Least Squares (OLS) regression models were fitted to the data to examine factors associated with AS-related work costs. The factors examined included the continuous variables age, AS function index (BASFI), and EQ5D score; and the binary variable, gender. In addition, nine cost indicators were considered as the dependent variables in the respective specifications. Logistic regression was used to examine retirement due to AS (Yes/No) and requirement for unpaid assistance (Yes/No) as the binary dependent variables. Seven multivariate OLS models were run where the dependent variables was patient-reported productivity loss index, ability score, difficulty score, cost due to absence from work, cost due to inefficient working hours at work (i.e. presenteeism), and the retirement gap (i.e. the difference in years between the usual age of retirement and the actual age at which the patients retired).

## Results

Of 570 patients invited, 482 (85%) returned completed questionnaires. Hospital records were available for all participants, while GP data was available for 150 participants at that time. Respondents were 77% male; the mean age was 55.5 years (SD±15.9); mean BASDAI 43 and BASFI 46.9. Mean disease duration was 19.8 years from diagnosis and 28.3 years from symptom onset. These results are consistent with demographics for other AS cohorts. The respondents’ demographic characteristics, stratified by employment status, are shown in Table 1.

**Table 1:** Demographic Characteristics of the study participants.

Characteristics	Non-working Patients	Working Patients
Number of patients	254	228
Number of Male (%)	193 (76)	167 (73)
Mean age in years (standard deviation)	59.74 (12.3)	46.73 (11.38)
Age<66 (% of patients)	169 (66.5)	215 (94.3)
Age retired (95% CI)	53.59 (52.14–55.04)	
Early retirement due to AS: No. of patients (% of patient)	173 (74)	
Gap (years) between usual & actual age of retirement *For patients with early retirement due to AS*	10.23 (8.59–11.89)	
Gap (years) between usual & actual age of retirement *For patients with early retirement not due to AS*	7.18 (4.69–9.66)	
*Mean comparison t-test p-value*	0.049	
Mean hours/week actually worked (95% CI) (n)		35.1 (32.93–37.29) n = 211
Mean hours/week absent due to AS (95% CI) (n)		
*For patients who were absent at least one hour*		7.61 (4.1–11.13) (n = 31)
*For all patients in the job*		1.15 (0.52–0.77) (n = 206)
	
Mean hours/week absent due to other reason (95% CI) (n)		
*For patients who were absent at least one hour*		13.26 (9.25–17.27) (n = 35)
*For all patients in the job*		2.20 (1.27–3.12) (n = 211)
	
Total hours/week working (95% CI) (n)		38.42 (36.52–40.32) (n = 211)
Productivity loss in daily regular activity (mean score on a 0–10 scale, higher values indicate higher loss)	5.22 (4.88–5.58) (n = 254)	3.28 (2.93–3.63) (n = 225)
Productivity loss in on-the-job work (mean score on a 0–10 scale, higher values indicate higher loss)		2.19 (1.89–2.50) (n = 223)
BASDAI: Mean (95% CI)	49.97 (46.79–53.15)	38.56 (35.47–41.66)
BASFI: Mean (95% CI)	58.29 (54.85–61.73)	35.13 (31.82–38.45)
Health Status: Mean (95% CI)	51.44 (48.51–54.37)	66.12 (63.48–68.76)
EQ5D: Mean (95% CI)	0.47 (0.43–0.52)	0.68 (0.65–0.71)
AS Duration from diagnosis: Years (95% CI)	23.59 (21.37–25.80)	15.60 (13.94–17.26)
AS Duration from first symptom Years (95% CI)	32.62 (30.47–34.78)	23.67 (21.98–25.37)

## Healthcare Resource Use

### GP Visits and GP-Related Events

The average number of visits is reported for all participants, while GP events are reported only for those patients whose information is present in the routine GP data (n = 150). Patients self-reported a mean of 1.73 (95%CI: 1.39–208) GP visits over the previous 3 month period compared to 1.35 (95%CI: 1.09–1.60) recorded in routine GP data for the same period. However, during the same 3 month recall period the routine GP data recorded a mean 6.11 (95%CI: 5.40–6.82) GP events for the participants, indicating substantial administrative costs even on non-visit days (S3 Table: Average number of GP visits and events for the AS patients from patient-derived data and routine data). The number of relevant prescriptions and GP procedures are shown in S4 Table (Drugs and Medications prescribed for AS patients from routine data) and S5 Table (GP Analysis: Healthcare resource procedures from routine data), respectively.

### GP and Prescription Costs

Two sets of cost estimates for GP utilisation and prescription costs are shown in Table 2 from patient-reported data and routine data. For patient-reported data, GP attendance and prescription costs were £1140 (95%CI: 935–1345) patient/year, mainly attributable to the use of DMARDs and anti-TNF drugs. GP costs using linked routine data are £1067 (95%CI: 955–1180) patient/year and include GP events (administration).

**Table 2:** General Practitioner (GP) and prescription costs for AS.

**Cost Items**	**All Patient**	**BASDAI Group**		**BASFI Group**		**Age**	
	**Mean (95% CI)**	**Mean (95% CI)**		**Mean (95% CI)**		**(Mean 95% CI)**	
		BASDAI < 40	BASDAI >40	BASFI < 40	BASFI >40	AGE < 50	AGE>50
	(n = 400)	(n = 188)	(n = 212)	(n = 175)	(n = 225)	(n = 150)	(n = 250)
Panel A: Estimates from Questionnaire Data							
(i) GP Visits	**241**	**185**	**290**	**176**	**291**	**209**	**260**
	(213–269)	(154–216)	(247–334)	(144–208)	(250–333)	(168–251)	(223–297)
(ii) GP prescription cost	**899**	**651**	**1119**	**500**	**1210**	**882**	**910**
	(698–1101)	(421–883)	(800–1438)	(295–706)	(894–1526)	(585–1178)	(640–1180)
*Painkiller and NSAID*	**82**	**45**	**116**	**55**	**104**	**67**	**92**
	(75–90)	(38–51)	(105–127)	(45–64)	(94–114)	(57–77)	(82–102)
*DMARDs and Anti-TNFs*	**817**	**607**	**1002**	**445**	**1106**	**815**	**818**
	(616–1018)	(377–838)	(683–1322)	(240–651)	(790–1421)	(518–1112)	(549–1088)
(iii) GP Travel costs	**11**	**8.7**	**13.4**	**8**	**13.7**	**13.4**	**9.9**
	(7.7–14.7)	(4.4–12.9)	(8.1–18.8)	(3.6–12.4)	(8.5–18.8)	(6.0–20.7)	(6.5–13.3)
**Total GP Cost (i + ii)**	**1140**	**837**	**1409**	**676**	**1501**	**1091**	**1170**
	(935–1345)	(599–1074)	(1087–1731)	(464–888)	(1182–1820)	(790–1391)	(894–1445)
Panel B: Estimates from Routine Data							
	**All Patient**	**BASDAI Group**		**BASFI Group**		**Age**	
		BASDAI < 40	BASDAI >40	BASFI < 40	BASFI >40	AGE < 50	AGE>50
	(n = 162)	(n = 72)	(n = 90)	(n = 71)	(n = 91)	(n = 74)	(n = 88)
(i) GP visits	**183**	**132**	**225**	**123**	**231**	**156**	**207**
	(156–210)	(100–163)	(185–265)	(94–152)	(190–271)	(118–193)	(168–246)
(ii) GP events (Administration)	**395**	**323**	**453**	**278**	**486**	**312**	**465**
	(352–438)	(264–381)	(394–512)	(227–329)	(427–545)	(256–368)	(405–524)
(iii) AS related Prescription	**489**	**343**	**605**	**301**	**635**	**400**	**563**
	(419–559)	(233–453)	(522–689)	(223–380)	(536–734)	(284–517)	(481–646)
**Total GP Cost (i + ii + iii)**	**1067**	**798**	**1283**	**703**	**1352**	**868**	**1235**
	(955–1180)	(632–963)	(1142–1424)	(573–832)	(1201–1502)	(694–1042)	(1095–1374)

Routine data GP prescription costs were £395 compared to £899/patient/year for self-report which can be attributed to the Welsh NHS funding model whereby anti-TNF agents are prescribed by secondary-care (hospital-based rheumatology clinics) and not by GPs (who prescribe all other medication).

### Hospital Costs

Consistent with the GP data, patient-reported estimates for hospital attendances (IP day unit, OP and A&E) were higher than those captured by routine data, and therefore associated with higher costs (S6 Table: Patient-reported and routine data estimates of outpatient, inpatient, and A&E attendance costs for AS patients (£/patient/year)). For both models, >95% of the hospital attendance costs were attributed to OP clinics and IP (which include infusion and day unit) episodes.

The average cost patient/year for consulting hospital-based healthcare professionals is £755, mostly incurred for consulting rheumatologists (35%; £267) and physiotherapists (23%; £174) (S7 Table: Distribution of healthcare cost stratified by disease severity, functional ability and age). Cost estimates for secondary care investigations are also shown in S7 Table, with the majority incurred for radiological investigations.

### Total NHS Cost

Using the above data, the total costs (£/patient/year) to the NHS (GP, hospital, prescription and investigation costs) were estimated at £3230 (95%CI 2666–3794) from patient-reported data and £2343 (95%CI 2090–2596) using routine alone (Table 3).

**Table 3:** Total NHS cost for AS stratified by disease severity, functional ability and age (£/patient/year).

Cost Items	All Patient	BASDAI Group		BASFI Group		Age	
	Mean (95% CI)	Mean (95% CI)		Mean (95% CI)		(Mean 95% CI)	
		BASDAI < 40	BASDAI >40	BASDFI < 40	BASFI >40	AGE < 50	AGE>50
	(n = 400)	(n = 188)	(n = 212)	(n = 175)	(n = 225)	(n = 150)	(n = 250)
**Panel A: Estimates from Questionnaire Data**							
Total Cost NHS	**3230**	**2384**	**3980**	**1994**	**4192**	**2811**	**3482**
	(2666–3794)	(1582–3187)	(3198–4763)	(1208–2780)	(3416–4969)	(1855–3768)	(2782–4181)
***Total Cost NHS (Lower bound estimate)***	***2803***	***2039***	***3481***	***1714***	***3650***	***2510***	***2979***
	(2374–3232)	(1468–2610)	(2859–4103)	(1160–2268)	(3040–4261)	(1792–3228)	(2442–3516)
***Total Cost NHS (Upper bound estimate)***	***3366***	***2494***	***4140***	***2083***	***4364***	***2909***	***364***
	***(2757–3976)***	*(1615–3373)*	*(3302–4978)*	*(1221–2945)*	*(3531–5197)*	*(1871–3946)*	*(2887–4395)*
**Panel B: Estimates from Routine Data**							
Total Cost NHS	**2343**	**1992**	**2654**	**1664**	**2871**	**1899**	**2609**
	(2090–2596)	(1659–2326)	(2282–3025)	(1374–1954)	(2495–3247)	(1594–2204)	(2251–2967)
***Total Cost NHS (Lower bound estimate)***	***2049***	***1757***	***2308***	***1514***	***2465***	***1746***	***2231***
	*(1871–2227)*	*(1524–1990)*	*(2046–2571)*	*(1311–1717)*	*(2202–2729)*	*(1498–1994)*	*(1989–2473)*
***Total Cost NHS (Upper bound estimate)***	***2447***	***2076***	***2775***	***1717***	***3014***	***1953***	***2743***
	*(2166–2727)*	*(1706–2445)*	*(2363–3188)*	*(1396–2039)*	*(2596–3432)*	*(1625–2281)*	*(2343–3143)*

### Factors Associated with Public Healthcare Costs

Patients with higher disease activity (BASDAI≥40) and higher functional impairment (BASFI≥40) incur significantly higher healthcare related costs than those with lower disease activity and better function. This group were also more likely to overestimate the number of self-reported GP visits (S3 Table). The marginal effects of function, disease activity and self-reported Health Status on cost are reported in Table 4. The coefficients indicate that a 10 unit increase in BASFI and BASDAI score are associated with £460 and £410 increases in the NHS costs, respectively, while a 10 unit decrease in health status is associated with a £540 increase in the NHS cost of AS.

**Table 4:** Marginal effect of BASFI, BASDAI, or Health Status on selected AS costs.

Covariates (Explanatory Variables)	Dependent Variables				
	NHS Cost	GP Cost	OP Cost	IP Cost	Out of Pocket Expense
**Panel A: Questionnaire Data**					
BASFI	**46.04** **	**14.32** **	**14.75** **	16.41*	**12.3** **
	(CI: 26.1, 66.0)	(CI: 7.1, 21.6)	(CI: 8.20, 21.3)	(CI: 0.04, 32.8)	(CI: 4.9, 19.8)
BASDAI	**40.64** **	**12.25** **	**16.33** **	**11.36** **	**14.1** **
	(CI: 18.2, 63.0)	(CI: 5.9,19.3)	(CI: 9.03, 23.6)	(CI: -6.9, 29.6)	(CI: 5.8, 22.4)
Health Status	**-54.41** **	**-17.13** **	**-18.01** **	**-18.07** **	**-23.1** **
	(CI: -78.9, -30.0)	(CI: -26.0, -8.3)	(CI: -26.0, -10.0)	(CI: -38.1, 2.0)	(CI: -32.1, -14.2)
**Panel B: Routine Data**					
BASFI	**25.1** **	10.7**	**6.9** **	9.7*	
	(CI: 11.5, 36.6)	(CI: 7.1, 14.3)	(CI: 3.7, 10.0)	(CI: 2.3, 17.1)	
BASDAI	**26.3** **	**9.4** **	**5.2** **	**7.6**	
	(CI: 11.0, 41.7)	(CI: 5.2, 13.6)	(CI: 1.6, 8.7)	(CI: -0.8, 15.9)	
Health Status	**-42.7**	**-14.5** **	**-9.6**	**-16.1** **	
	(CI:- 59.4, -25.9)	(CI: -19.0, -10.0)	(CI: -13.4, -5.8)	(CI: -25.2, -7.1)	

Note: 95% confidence intervals for the coefficients are reported in the parentheses.

** implies that coefficients are significant at 1% level of significance (* implies 5% level). For each covariate, the regressions were run separately controlling for age and sex. In the above table the marginal effects are therefore generated from running 15 specifications. Age was a significant determinant for IP costs.

### Out-of-Pocket AS-Related Costs

The mean AS-related, out-of-pocket expenses incurred by participants was £705/person/year while one-off purchase expenses were £362/person/year (S8 Table: Out of Pocket AS-related costs for the AS Patients (£/AS patient/year)). These costs include £58/person/year for transport to healthcare appointments and £113/person/year for self-funded exercise outside of the NHS.

## Work Productivity Loss

### Employment and Early Retirement

At the time of responding, 53% (254/482) of participants were not working (See Table 1). Of the total 397 participants of working age (<65 years), 43% (169/397) were not working, which entails a 57% labour force participation rate for AS patients. Of those no longer working, the mean age of retirement was 53.6 years, with 74% citing AS as the main reason for early retirement. On average, the AS patients retired 9.5 years earlier than the general population and 10.2 years earlier than the usual retirement age for their specific occupation.

### Absenteeism and Presenteeism

AS impacts both on the ability to attend work (absenteeism) and productivity while at work (presenteeism). Participants reported the impact of AS on work performance (S9 Table: Impact of AS on on-the-job performance due to physical and emotional problems). On average, participants missed 3.5% (95%CI: 1.6–5.3, n = 197) of their work time (absenteeism) (Table 5). In addition, they report 21.6% (95%CI: 18.6–24.7, n = 225) impairment of working level (presenteeism) and 26.1% (95%CI: 22.7–29.6, n = 197) overall work impairment due to AS.

**Table 5:** Work productivity and activity impairment outcomes as assessed by the Work Productivity Impairment Questionnaire.

Work Productivity Cost Components	All Patients	BASDAI Group		BASFI Group	
**Working AS Patients** (%; 95%CI; n)					
Percent work time missed due to health	**3.46**	**1.43**	**5.69**	**1.1**	**7.5**
	(CI: 1.6–5.3)	(CI: 0.54–2.32)	(CI: 1.92–9.46)	(CI: 0.37–1.74)	(CI: 2.7–12.2)
	(n = 197)	(n = 103)	(n = 94)	(n = 123)	(n = 74)
Percent impairment while working due to health	**21.6**	**11.7**	**33.4**	**13.9**	**33.5**
	(CI: 18.6–24.7)	(CI: 9.2–14.3)	(CI: 28.2–38.6)	(CI: 11.5–16.3)	(CI: 27.4–39.6)
	(n = 225)	(n = 122)	(n = 103)	(n = 136)	(n = 89)
Percent overall work impairment due to health	**26.1**	**15**	**38.3**	**16.2**	**42.7**
	(CI: 22.7–29.6)	(CI: 12.0–18.0)	(CI: 32.9–43.8)	(CI: 13.6–18.8)	(CI: 36.2–49.2)
	(n = 197)	(n = 103)	(n = 94)	(n = 123)	(n = 74)
Percent activity impairment due to health	**32.8**	**22.6**	**44.9**	**22.9**	**48**
	(CI: 29.3–36.3)	(CI: 18.8–26.4)	(CI: 39.6–50.1)	(CI: 19.5–26.3)	(CI: 42.1–53.8)
	(n = 225)	(n = 122)	(n = 103)	(n = 136)	(n = 89)
**AS Patients not at Work** (%; 95%CI; n)					
Productivity losses in daily regular activities	**52.3**	**33.1**	**63.1**	**24.5**	**62.5**
	(CI: 49.1–56.0)	(CI: 27.6–38.6)	(CI: 59.5–66.7)	(CI: 18.7–30.4)	(CI: 59.3–65.7)
	(n = 253)	(n = 89)	(n = 164)	(n = 66)	(n = 187)

Overall activity impairment due to ill-health was 33% for those in work (work and home activities) and 52% for those not in work (home). Higher disease activity and functional impairment were associated with significantly increased productivity and activity impairment (Table 5).

Of employed participants, 13.6% (31/228) were absent from work during the past 7 days due to AS and missed on average 7.6 (95%CI: 4.36–10.86) hours of work. For these participants the self-reported mean productivity loss was 2.2 (CI: 1.9–2.5) indicating more than 20% loss of productivity during the working hours. The productivity impact on daily regular household tasks was 3.3 (i.e. more than 30% loss of productivity).

### Work-Related Costs of AS

Using the age and sex specific national average daily wage rates (S2 Table: Hourly pay rate, gross (£) for all employee jobs in the United Kingdom at 2010 prices), annual losses to earnings per patient (not in paid work) were £23691 (95%CI: 21849–25534] under the human capital approach (cost to patient). Spread across all AS patients in the cohort (working and not working), the early retirement cost of AS was estimated to be £8107 (95%CI: 6834–9380) year/patient, and was significantly higher in those with BASDAI and BASFI ≥40, but there were no significant gender differences (Table 6).

**Table 6:** Work productivity loss cost estimates relating to early retirement, absenteeism, and presenteeism.

Cost Components	All Patients	BASDAI Group		BASFI Group		Sex	
	Mean (95% CI) (n)	Mean (95% CI) (n)		Mean (95% CI) (n)			
		BASDAI < 40	BASDAI >40	BASFI < 40	BASFI >40	Female	Males
**Early retirement** Cost(£)/year/AS patients	**8107**	**5765**	**9961**	**3740**	**11284**	**9459**	**7849**
	(CI: 6834–9380)	(CI: 4106–7423)	(CI: 8117–11806)	(CI: 2313–5168)	(CI: 9425–13142)	(CI: 6928–11990)	(CI: 6172–9125)
	(n = 482)	(n = 213)	(n = 269)	(n = 203)	(n = 279)	(n = 122)	(n = 360)
**Absenteeism** Cost(£)/year/AS patients	**411**	**170**	**602**	**158**	**595**	**610**	**344**
	(CI: 183–639)	(CI: 55–286)	(CI: 204–1000)	(CI: 51–265)	(CI: 209–981)	(CI: 85–1135)	(CI: 94–593)
	(n = 482)	(n = 213)	(n = 269)	(n = 203)	(n = 279)	(n = 122)	(n = 360)
**Presenteeism** (Cost(£)/year/AS patients)	**3425**	**2300**	**4316**	**3259**	**3546**	**3387**	**3438**
	(CI: 2823–4028)	(CI: 1695–2905)	(CI: 3359–5274)	(CI: 2524–3994)	(CI: 2650–4443)	(CI: 2232–4542)	(CI: 2730–4147)
	(n = 482)	(n = 213)	(n = 269)	(n = 203)	(n = 279)	(n = 122)	(n = 360)
*Presenteeism (Cost(£)/year/working patients)*	**7241**	**3950**	**11165**	**4829**	**10872**	**6774**	**7412**
	(CI: 6163–8319)	(CI: 3010–4891)	(CI: 9336–12993)	(CI: 3840–5818)	(CI: 8808–12936)	(CI: 4782–8765)	(CI: 6121–8703)
	n = 228	(n = 124)	(n = 104)	(n = 137)	(n = 91)	(n = 61)	(n = 167)
**Total (Absenteeism + Presenteeism)** (Cost(£)/year/AS patients)	**3836**	**2470**	**4918**	**3417**	**4141**	**3997**	**3782**
	(CI: 3131–4542)	(CI: 1840–3100)	(CI: 3769–6068)	(CI: 2666–4169)	(CI: 3049–5234)	(CI: 2628–5366)	(CI: 2955–4609)
	(n = 482)	(n = 213)	(n = 269)	(n = 203)	(n = 279)	(n = 122)	(n = 360)
***Total (Absenteeism + Presenteeism)*** *(Cost(£)/year/* ***working patients*** *)*	**8110**	**4243**	**12721**	**5063**	**12697**	**7993**	**8153**
	(CI: 6825–9395)	(CI: 3270–5215)	(CI: 10434–15009)	(CI: 4058–6069)	(CI: 10100–15294)	(CI: 5630–10357)	(CI: 6612–9694)
	(n = 228)	(n = 124)	(n = 104)	(n = 137)	(n = 91)	(n = 61)	(n = 167)

The work-related AS costs for those patients in employment, was estimated to be £869 (95%CI: £392–1346) due to lost hours at work (absenteeism) and £7241 (95%CI: 6163–8319) due to the inefficient hours while working (presenteeism) per year per working patient. Spread across the entire cohort (working and non-working), the estimates were £411 for absenteeism and £3425 for presenteeism per year per AS patient (Table 6). Costs for absenteeism was associated with higher disease activity and higher functional impairment, while presenteeism was mainly associated with higher disease activity (Table 6 & S10 Table 10: Determinants of AS related work productivity loss costs).

### Factors Associated with Work Productivity Loss

Using logistic regression, early retirement due to illness was associated with functional impairment, while using linear regression productivity loss (work and home) was associated with younger age, increased functional impairment and lower quality of life (S10 Table). While the costs due to absenteeism and presenteeism were associated with functional impairment, neither was associated with gender. In addition, costs for absenteeism due to AS were associated with lower age, while costs for presenteeism were associated with lower quality of life.

### Cost of Unpaid Assistance

Patients with chronic diseases often require significant assistance from unpaid carers (usually family members) who may themselves incur costs as a result of this (e.g. time off work for carer-related activities). AS patients required an average of 52 hours of unpaid care givers’ time during a 3 month period, incurring costs of £1279 (95%CI 903–1655) and £2983 (95%CI 2105–3680) year/patient when estimated using the minimum and mean national wage, respectively (S11 Table: Cost estimates of unpaid assistance). The cost incurred due to unpaid assistance and accompaniment for visits to various healthcare facilities was £193 (95%CI 91–295) year/patient at mean national wage. Men have less assistance from informal carers than women (S11 Table: Cost estimates of unpaid assistance).

### Total Combined Costs

From the data presented above, it is clear that neither patient-reported nor routine data costs alone can accurately capture the full costs of AS. We therefore calculated total costs by combining the datasets that most accurately capture the real-life situation for AS in the UK. Using these datasets, the total cost of AS is estimated to be in the region of £19016 (95%CI: 14854–23149)/patient/year (Table 7). This is calculated using routine datasets for GP attendance visits (as it appears that patient-reported visits are an overestimate), GP administration events (not captured at all in patient-reported data) and hospital (outpatient, inpatient and A&E) costs from routine data (as for GP visits), while patient-reported questionnaires were used for prescription costs (as medications like anti-TNF are not captured in the GP dataset), and self-funded non-NHS costs; out-of-pocket expenses, one-off purchase expense, cost of transport to health professionals, early retirement, absenteeism and presenteeism (patient-reported) and unpaid assistance costs.

**Table 7:** Combined total cost of AS (£/AS patient/year).

Cost items	All Patient Mean (95% CI) (n = 400)
**GP visits/attendance** *	**183 (156–210)**
**Prescription costs**	**899 (698–1101)**
**GP events (administration)** *	**395 (352–438)**
**Outpatient costs** *	**558 (469–646)**
**In-patient costs** * **(incl. day unit)**	**710 (503–917)**
**A&E visit costs** *	**8.1 (4.2–12.0)**
**Non-NHS therapies (self-funded)**	**61 (48–74)**
**Out of pocket expenses + one off purchase**	**1067 (668–1526)**
**Transport to health care professionals**	**16.3 (10.3–22.8)**
**Early retirement**	**8107 (6834–9380)**
**Absenteeism**	**411 (183–639)**
**Presenteeism**	**3425 (2823–4028)**
**Cost of unpaid assistance at mean wage**	**2983 (2015–3860)**
**Cost of unpaid assistance for health care visits at mean wage**	**193 (91–295)**
**Total cost**	**£19,016 (14,854–23,149)**

Note: *indicates costs calculated from routine datasets. Other costs are calculated from patient-reported questionnaires.

## Discussion

We examine the total costs associated with AS in the UK using a population based cohort to capture linked routine and patient-reported data. We employed the human capital approach so productivity costs are included and therefore costs are not underestimated, as may be the case for the friction cost approach. We found that patients with AS incur significant costs as a result of their condition. In particular, AS has a significant effect on the ability to work with 43% of people of working age either unemployed or retired early, and ≥70% citing AS as the cause. We estimated that the total cost of AS is £19016/person/ year including NHS costs, patient costs and society costs. However, the majority of the costs are due to early retirement, inefficient working and unpaid carers’ time. Therefore, much of the costs of AS are hidden and not captured using traditional methods. The cost of AS varies greatly with function and age.

The combination of patient-reported and linked healthcare data used here offers the opportunity to capture the most accurate and comprehensive dataset for cost calculations. This methodology has allowed us to demonstrate that patients appear to overestimate their NHS health-care visits in comparison to routine GP and hospital administrative records for the same period. Routine data also allows GP administration costs to be captured, which are not available elsewhere. Therefore, linked routine data appears to be the most accurate in capturing healthcare visits in our setting. In contrast, effective and expensive AS medications such as TNF-inhibitors are prescribed in specialist secondary-care settings in the UK, and therefore not captured in the current routine GP prescribing dataset (these may be available in free-text fields to which we do not have access for reasons of data privacy). Therefore, patient-reported data is vital for accurate records of medications in the absence of a comprehensive prescriptions register. Recall is also less of an issue here as patients have written prescriptions from their GPs and are unlikely to forget that they receive injectable TNF-inhibitors for their AS. Thus, the novel combination of both patient and linked routine data provides the optimal information for estimating AS-related healthcare costs.

Our findings are comparable with previous UK research which also found that 45% of those of working age were unemployed, 20% were work impaired and there was 14.9% AS-related absenteeism, missing 8.78 days over a three month period (1). Our study finds a cost at £3836 due to loss of work for employed people and £8107 due to early retirement which equates to £11943 per year per patient. This compares with £2342 per 3 months or £9368 per year reported by Rafia et al [1]. Thus, findings from both studies for work-related costs are very similar and the small differences might be explained by a difference in number of people in work in Wales compared to England and differences in the populations studied.

Another UK study utilising secondary care medical records in 2008 reported costs attributable to IP (£382), OP (£306), physiotherapy (£598) and medication (£372)(9). Our equivalent figures today would give IP (£710), OP (£558), physiotherapy (£174) and medication (£899). Our data suggests that, compared to this earlier study, there is a trend for reduced physiotherapy and increased OP and IP attendance and costs. This is consistent with our observations in clinical practice, since the introduction of TNF-inhibitors prescribed for AS, which have increased the requirement for specialist (rheumatology) hospital-based input and reduced the need for physiotherapy, due to better outcomes. Our estimated patient-reported medication costs were higher (£899) which reflects the introduction of expensive anti-TNF therapy (used in 15% of our cohort, which is comparable to 20% in another UK study [38]) for the treatment of AS since 2008 and the fact that we also captured primary care medication.

Our findings enhance existing evidence that impaired function and increased disease activity lead to significant increase in the cost of AS. Therefore, interventions that improve the function and work productivity of patients are likely to lead to the largest reduction in the costs of AS which are dominated by work-related costs.

This study gives the most comprehensive examination of the cost of AS in the UK, ever. However, as with all economic evaluations, there are some limitations. Questionnaires could be completed via postal or electronic methods, as such the equivalence of data from the two different formats may have been an issue [39]. However, care was taken to avoid any substantial changes when migrating the paper questionnaire to electronic format in order to reduce response bias.

Visits to GPs are difficult to accurately identify using routine data, while patients tend to over-report visits to health professionals. Future work could obtain the attendance log at a sample of GP practices to develop algorithms which accurately identify GP visits. Although this work is the most comprehensive published evaluation of the costs of AS in the UK, there are nevertheless still some costs which are not captured. For example, prescriptions through secondary care are not captured using GP data and are not currently available for these patients, however patient-reported prescriptions give a proxy to this cost. Similarly, hospital administration costs which are likely to be considerable due to the administration associated with biologics, are not currently available and as such, costs will be underestimated here. Cost analysis has been calculated at 2010 prices and so today’s cost would be increased.

At the time of study, measures of psychological well-being were not available for analysis, however, research has shown that depression is associated with employment, absenteeism and presenteeism[40]].

We have produced the most comprehensive cost analysis of AS in the UK to-date, using routinely collected data in conjunction with patient-reported data that allows the hidden costs, such as presenteeism, absenteeism and early retirement when calculating work productivity loss costs and the effect of disease severity on cost estimates for the condition. This methodology can also be adapted to develop enhanced health-economic analyses for a range of chronic, long-term conditions.

## Conclusions

The total cost of AS in the UK at the time of this work is estimated to be £19,016 per person per year and is significantly higher in patients with poor function. The major cost of AS is as a result of inefficient working hours, early retirement and in unpaid carer’s time. Interventions which can keep people in productive work until retirement age would have the greatest single impact in reducing costs in AS.

## Supporting Information

S1 Table Unit costs. (DOCX)

S2 Table Hourly pay rate, gross (£) for all employee jobs in the United Kingdom at 2010 prices. (DOCX)

S3 Table Average number of GP visits and events for the AS patients from patient-derived data and routine data. (DOCX)

S4 Table Drugs and Medications prescribed for AS patients from routine data. (DOCX)

S5 Table GP Analysis: Healthcare resource procedures from routine data. (DOCX)

S6 Table Patient-reported and routine data estimates of outpatient, inpatient, and A&E attendance costs for AS patients (£/patient/year). (DOCX)

S7 Table Distribution of healthcare cost stratified by disease severity, functional ability and age. (DOCX)

S8 Table Out of Pocket AS-related costs for the AS Patients (£/AS patient/year). (DOCX)

S9 Table Impact of AS on on-the-job performance due to physical and emotional problems as assessed by the Work Limitations Questionnaire. (DOCX)

S10 Table Determinants of AS related work productivity loss costs. (DOCX)

S11 Table Cost estimates of unpaid assistance. (DOCX)
