# Supplementary material for: The Cost of Ankylosing Spondylitis in the UK Using Linked Routine and Patient-Reported Survey Data
Source: PLoS One. 2015 Jul 17;10(7):e0126105. doi: 10.1371/journal.pone.0126105 (PMC4506082; doi:10.1371/journal.pone.0126105)
Supplement: S1 Table — (DOCX) [file pone.0126105.s001.docx]

**Supplementary** **Table 1: Unit Costs**

| **Item #** | **Cost Components** | **Unit Cost** | **Unit** | **Source** |
| --- | --- | --- | --- | --- |
| 1 | GP visit (consultations) | 36 | £/Per clinic consultation lasting 11.7 minutes | Unit Costs of Health and Social Care 2010 (P.167) |
| 2 | GP event (administration) | 22 | Equivalent of per telephone consultation lasting 7.1 minutes | Unit Costs of Health and Social Care 2010 (P.167) |
| 3 | Average Prescription Cost | 39 | £/Prescription costs per consultation (actual cost) | Unit Costs of Health and Social Care 2010 (P.167) |
| 4 | Outpatient attendance | 137 | £/outpatient attendance | National Schedule of Reference Costs Year : 2010-11 - NHS Trusts and PCTs combined Outpatient Attendances Data |
| 5 | Inpatient Admission – national average (Lower Quartile; Upper Quartile) | 2644  (1665; 2937) | £/inpatient episodes | National Schedule of Reference Costs Year : 2010-11 - NHS Trusts and PCTs combined Elective Inpatient HRG Data |
| 6 | Inpatient Day cases national average (Lower Quartile; Upper Quartile) | 542  (319; 620) | £/inpatient day | National Schedule of Reference Costs Year : 2010-11 - NHS Trusts and PCTs combined Elective Inpatient HRG Data |
| 7 | Accidents and Emergencies attendance (Lower Quartile: Upper Quartile) | 202  (158; 238) | A&E treatments leading to admitted | National Schedule of Reference Costs Year : 2010-11 - NHS Trusts and PCTs combined Accident and Emergency Services: Leading to Admitted |
| 8 | Transport cost – car | 0.205 | £/mile | AA Motoring cost 2010 (Running cost pence/mile) |
| 9 | Transport cost – Bus | 0.08 | £/mile | <http://www.guardian.co.uk/flash/0,5860,632634,00.html> |
| 10 | Transport cost - train | 0.3 | £/mile | <http://www.guardian.co.uk/flash/0,5860,632634,00.html> |
| 11 | Transport cost - train | 15 | £/return travel | Assumed flat rate |
| 12 | Transport cost - other | 12 | £/visit | Based on NHS travel allowance scheme |
| 13 | Visit to Rheumatologist | 132 | £/attendance | National Schedule of Reference Costs Year : 2010-11 - NHS Trusts Consultant Led: Follow up Attendance Non-Admitted Face to Face |
| 14 | Visit to Physiotherapist | 47 | £/attendance | National Schedule of Reference Costs Year : 2010-11 - NHS Trusts Consultant Led: Follow up Attendance Non-Admitted Face to Face |
| 15 | Visit to Radiologist | 114 | £/attendance | Unit Costs of Health and Social Care 2010; p.199 |
| 16 | Visit to Chiropractor /Osteopath | 36 | £/attendance | Unit Costs of Health and Social Care 2010; p.154 |
| 17 | Visit to nurse | 41 | £/attendance | Unit Costs of Health and Social Care 2010 p.208 |
| 18 | Visit to Rheumatology specialist nurse | 77 | £/attendance | Unit Costs of Health and Social Care 2010 p.162 |
| 19 | Visit to health visitor | 42 | £/attendance | Unit Costs of Health and Social Care 2010 p.161 |
| 20 | Visit to Optometrist | 54 | £/attendance | National Schedule of Reference Costs Year : 2010-11 - NHS Trusts Outpatient Attendances Data |
| 21 | Visit to ophthalmologist | 83 | £/attendance | National Schedule of Reference Costs Year : 2010-11 - NHS Trusts Outpatient Attendances Data |
| 22 | Visit to Psychologist | 81 | £/attendance | Unit Costs of Health and Social Care 2010 p.155 |
| 23 | Visit to counsellor | 41 | £/attendance | National Schedule of Reference Costs Year : 2010-11 - NHS Trusts Outpatient Attendances Data |
| 24 | Visit to occupational therapist | 64 | £/attendance | National Schedule of Reference Costs Year : 2010-11 - NHS Trusts Outpatient Attendances Data |
| 25 | Visit to other | 65 |  | Average of others |
| 26 | Cost of Bone density scan (DXA) | 72 | £/imaging | National Schedule of Reference Costs Year : 2010-11 - NHS Trusts Diagnostic Imaging: Outpatient |
| 27 | Cost of Radio-Active Bone Scan | 188 | £/imaging | National Schedule of Reference Costs Year : 2010-11 - NHS Trusts Diagnostic Imaging: Outpatient (Average of all imaging items) |
| 28 | Cost of X-Ray | 72 | £/imaging | Same as Dexa Scan |
| 29 | Cost of MRI | 243 | £/imaging | National Schedule of Reference Costs Year : 2010-11 - NHS Trusts Diagnostic Imaging: Outpatient (average of all type) |
| 30 | Cost of CT Scan | 122 | £/imaging | National Schedule of Reference Costs Year : 2010-11 - NHS Trusts Diagnostic Imaging: Outpatient (average of all type) |
| 31 | Cost of Blood Test | 11 | £/test | National Schedule of Reference Costs Year : 2010-11 - NHS Trusts Direct Access: Pathology Services |
| 32 | Cost of Urine Test | 11 | £/test | National Schedule of Reference Costs Year : 2010-11 - NHS Trusts Diagnostic Imaging: Outpatient (average of all type) |
| 33 | Cost of Gastroscopy | 188 | £/test | National Schedule of Reference Costs Year : 2010-11 - NHS Trusts Diagnostic Imaging: Outpatient (average of all type) |
| 34 | Cost of other type of test | 11 | £/test | National Schedule of Reference Costs Year : 2010-11 - NHS Trusts Diagnostic Imaging: Outpatient (average of all type) |
| 35 | Cost of Physiotherapy | 42 | £/per hour of client contact | Unit Costs of Health and Social Care 2010 p.151 |
| 36 | Cost of Hydrotherapy | 42 | £/therapy | Unit Costs of Health and Social Care 2010 p.152 |
| 37 | Cost of Occupational Therapy | 68 | £/therapy | Unit Costs of Health and Social Care 2010 p.152 |
| 38 | Cost of Counselling | 41 | £/therapy | Unit Costs of Health and Social Care 2010 |
| 39 | Cost of other type of consultation | 41 | £/therapy | Unit Costs of Health and Social Care 2010 |
| 40 | Paracetamol | 2.85 | £/item | Prescription cost for Wales 2010 (average price) |
| 41 | Other Painkillers | 10.63 | £/item | Prescription cost for Wales 2010 (average price) |
| 42 | Ibuprofen | 10.28 | £/item | Prescription cost for Wales 2010 (average price) |
| 43 | Indomethacin | 7.74 | £/item | Prescription cost for Wales 2010 (average price) |
| 44 | Naproxen (and similar) | 3.90 | £/item | Prescription cost for Wales 2010 (average price) |
| 45 | Other Anti-inflammatory/NSAIDs | 7.30 | £/item | Prescription cost for Wales 2010 (Weighted average) |
| 46 | Sulfasalazine | 25.35 | £/item | Prescription cost for Wales 2010 (average price) |
| 47 | Methotrexate | 4.92 | £/item | Prescription cost for Wales 2010 (average price) |
| 48 | Infliximab | 419 | £/item | Prescription cost for Wales 2010 (average price) |
| 49 | Etanercept (Enbrel) | 580 | £/item | Prescription cost for Wales 2010 (average price) |
| 50 | Adalimumab | 596 | £/item | Prescription cost for Wales 2010 (average price) |
| 51 | Other medications | 60.60 | £/item | Prescription cost for Wales 2010 (weighted average based on patients’ usage of the above-mentioned drugs) |
| 52 | AS related prescription cost | 60.60 | £/prescription | Prescription cost for Wales 2010 (weighted average price) |
