# Supplementary material for: The Cost of Ankylosing Spondylitis in the UK Using Linked Routine and Patient-Reported Survey Data
Source: PLoS One. 2015 Jul 17;10(7):e0126105. doi: 10.1371/journal.pone.0126105 (PMC4506082; doi:10.1371/journal.pone.0126105)
Supplement: S2 Table — (DOCX) [file pone.0126105.s002.docx]

Supplementary Table 2: Hourly pay rate, gross (£) for all employee jobs in the United Kingdom at 2010 prices

| **Description** | **Mean** |
| --- | --- |
| All employees | 14.60 |
| 16-17 | 5.12 |
| 18-21 | 7.32 |
| 22-29 | 11.36 |
| 30-39 | 15.59 |
| 40-49 | 16.45 |
| 50-59 | 15.69 |
| 60+ | 13.51 |

**Source:** Annual Survey of Hours and Earnings, Office for National Statistics.
