# Supplementary material for: The Cost of Ankylosing Spondylitis in the UK Using Linked Routine and Patient-Reported Survey Data
Source: PLoS One. 2015 Jul 17;10(7):e0126105. doi: 10.1371/journal.pone.0126105 (PMC4506082; doi:10.1371/journal.pone.0126105)
Supplement: S3 Table — (DOCX) [file pone.0126105.s003.docx]

Supplementary Table 3: Average number of GP visits and events for the AS patients from patient-derived data and routine data

|  | **Time frames for Data Sets** | **All Patient**  Mean  (95% CI) (n) | **BASDAI Group** | | **BASFI Group** | | **AGE Group** | |
| --- | --- | --- | --- | --- | --- | --- | --- | --- |
|  |  |  | **BASDAI<40**  Mean  (95% CI) (n) | **BASDAI≥40**  Mean  (95% CI) (n) | **BASFI<40**  Mean  (95% CI) (n) | **BASFI≥40**  Mean  (95% CI) (n) | **Age<50**  Mean  (95% CI) (n) | **Age≥50**  Mean  (95% CI) (n) |
| **Average number of GP Visits from questionnaire** | 3 months Recall Period | **1.73**  (1.39-2.08) (146) | **1.19**  (0.90-1.47) (64) | **2.16**  (1.60-2.72) (82) | **1.13**  (0.83-1.43) (62) | **2.18**  (1.64-2.72) (84) | **1.49**  (1.05-1.93) (63) | **1.92**  (1.41-2.42) (83) |
| **Average number of GP visits from routine data** | 3 months Recall Period | **1.35**  (1.09-1.60) (150) | **1.09**  (0.73-1.45) (65) | **1.54**  (1.18-1.90) (85) | **1.08**  (0.73-1.42) (64) | **1.55**  (1.18-1.91) (86) | **1.22**  (0.83-1.60) (65) | **1.45**  (1.10-1.80) (85) |
|  | 1 year retrospective | **4.96**  (4.22-5.69) (162) | **3.56**  (2.70-4.41) (72) | **6.08**  (5.00-7.16) (90) | **3.32**  (2.54-4.11) (71) | **6.23**  (5.14-7.32) (91) | **4.20**  (3.20-5.20) (74) | **5.59**  (4.54-6.64) (86) |
|  | 5 year retrospective | **20.13**  (17.5-22.8) (176) | **15.19**  (11.7-18.7) (80) | **24.24**  (20.5-28.0) (92) | **12.35**  (9.90-14.8) (80) | **26.60**  (22.6-30.6) (96) | **16.08**  (12.4-19.8) (84) | **23.82**  (20.1-27.5) (92) |
|  | 6 months prospective | **2.64**  (2.23-3.04) (158) | **2.10**  (1.55-2.65) (68) | **3.04**  (2.47-3.62) (90) | **1.83**  (1.33-2.32) (69) | **3.27**  (2.69-3.85) (89) | **2.22**  (1.70-2.75) (71) | **2.98**  (2.38-3.58) (85) |
| **Average number of GP events from routine data** | 3 months Recall Period | **6.11**  (5.40-6.82) (150) | **4.85**  (4.03-5.66) (65) | **7.07**  (6.03-8.11) (85) | **4.41**  (3.63-5.18) (64) | **7.37**  (6.36-8.39) (86) | **5.14**  (4.16-6.12) (65) | **6.85**  (5.87-7.82) (85) |
|  | 1 year retrospective | **22.9**  (20.5-25.3) (162) | **18.22**  (15.1-21.3) (72) | **26.66**  (23.3-30.0) (90) | **15.97**  (13.3-18.7) (71) | **28.32**  (25.0-31.6) (91) | **18.38**  (15.2-21.6) (74) | **26.72**  (23.4-30.0) (86) |
|  | 5 year retrospective | **88.86**  (79.7-98.0) (176) | **71.88**  (58.8-85.0) (80) | **103.01**  (90.7-115.2) (92) | **59.35**  (48.8-69.9) (80) | **113.45**  (101-126) (96) | **68.5**  (56.3-80.7) (84) | **107.45**  (94.8-120) (92) |
|  | 6 months prospective | **12.47**  (11.00-14.0) () | **10.24**  (8.20-12.3) (68) | **14.17**  (12.1-16.2) (90) | **8.64**  (6.90-10.4) (69) | **15.45**  (13.4-17.5) (89) | **9.86**  (7.97-11.75) (71) | **14.61**  (12..5-16.7) (85) |
