# Supplementary material for: The Cost of Ankylosing Spondylitis in the UK Using Linked Routine and Patient-Reported Survey Data
Source: PLoS One. 2015 Jul 17;10(7):e0126105. doi: 10.1371/journal.pone.0126105 (PMC4506082; doi:10.1371/journal.pone.0126105)
Supplement: S4 Table — (DOCX) [file pone.0126105.s004.docx]

Supplementary Table 4: Drugs and Medications prescribed for AS patients from routine data

| **Drug Group** | **Time frames for Routine Data Sets** | **All Patient**  Mean (95% CI) (n) | **BASDAI**  Mean (95% CI) (n) | | **BASFI**  Mean (95% CI) (n) | | **AGE**  Mean (95% CI) (n) | |
| --- | --- | --- | --- | --- | --- | --- | --- | --- |
|  |  |  | **BASDAI<40** | **BASDAI≥40** | **BASFI<40** | **BASFI≥40** | **Age<50** | **Age≥50** |
| **Total number of prescriptions** | 3 months Recall Period | **3.13**  (2.75-3.52) (150) | **2.66**  (2.11-3.21) (65) | **3.49**  (2.96-4.03) (85) | **2.30**  (1.85-2.75) (64) | **3.76**  (3.20-4.31) (86) | **2.57**  (2.01-3.13) (65) | **3.56**  (3.05-4.08) (85) |
|  | 1 year retrospective | **11.94**  (10.57-13.30) (162) | **9.39**  (7.28-11.50) (72) | **13.98**  (12.3-15.7) (90) | **7.99**  (6.42-9.55) (71) | **15.02**  (13.1-16.9) (91) | **9.07**  (6.98-11.16) (74) | **14.35**  (12.7-16.0) (88) |
|  | 5 year retrospective | **45.88**  (39.90-51.87) (176) | **35.71**  (26.9-44.6) (80) | **54.35**  (46.5-62.2) (96) | **27.38**  (21.1-33.6) (80) | **61.30**  (52.7-69.9) (96) | **32.96**  (24.4-41.6) (84) | **57.67**  (50.0-65.4) (92) |
|  | 6 months prospective | **6.08**  (5.26-6.89) (158) | **4.88**  (3.64-6.12) (68) | **6.98**  (5.91-8.04) (90) | **4.09**  (3.15-5.02) (69) | **7.62**  (6.45-8.79) (89) | **4.48**  (3.39-5.57) (71) | **7.38**  (6.25-8.51) (87) |
| **AS related Drugs** | 3 months Recall Period | **2.19**  (1.86-2.52) (150) | **1.68**  (1.15-2.21) (65) | **2.59**  (2.17-3.00) (85) | **1.53**  (1.12-1.94) (64) | **2.69**  (2.22-3.15) (86) | **1.86**  (1.32-2.40) (65) | **2.45**  (2.03-2.86) (85) |
|  | 1 year retrospective | **8.07**  (6.92-9.22) (162) | **5.67**  (3.85-7.48) (72) | **9.99**  (8.61-11.3) (90) | **4.97**  (3.67-6.27) (71) | **10.48**  (8.85-12.41) (91) | **6.61**  (4.69-8.52) (74) | **9.30**  (7.94-10.7) (88) |
|  | 5 year retrospective | **31.41**  (26.65-36.18) (176) | **38.53**  (32.7-44.4) (80) | **22.88**  (15.4-30.4) (96) | **17.85**  (13.0-22.7) (80) | **42.72**  (35.7-49.7) (96) | **23.74**  (16.4-31.0) (84) | **38.42**  (32.4-44.4) (92) |
|  | 6 months prospective | **4.00**  (3.38-4.62) (158) | **2.90**  (1.92-3.88) (68) | **4.84**  (4.07-5.62) (90) | **2.64**  (1.91-3.36) (69) | **5.07**  (4.17-5.97) (89) | **3.07**  (2.10-4.04) (89) | **4.77**  (3.98-5.56) (87) |
| **Musculoskeletal and Joint drugs** | 3 months Recall Period | **1.03**  (0.82-1.24) (150) | **0.86**  (0.56-1.16) (65) | **1.15**  (0.86-1.45) (85) | **1.03**  (0.69-1.37) (64) | **1.02**  (0.75-1.29) (86) | **1.11**  (0.78-1.44) (65) | **0.96**  (0.69-1.24) (85) |
|  | 1 year retrospective | **3.91**  (3.20-4.63) (162) | **3.01**  (2.00-4.03) (72) | **4.63**  (3.64-5.63) (90) | **3.41**  (2.33-4.49) (71) | **4.31**  (3.33-5.28) (91) | **3.96**  (2.86-5.06) (74) | **3.87**  (2.91-4.84) (88) |
|  | 5 year retrospective | **16.39**  (13.53-19.24) (176) | **19.57**  (15.5-23.6) (80) | **12.56**  (8.64-16.5) (96) | **12.29**  (8.51-16.03) (80) | **19.80**  (15.7-23.9) (96) | **14.29**  (10.3-18.3) (84) | **18.30**  (14.2-22.4) (92) |
|  | 6 months prospective | **1.93**  (1.53-2.33) (158) | **1.41**  (0.89-1.93) (68) | **2.32**  (1.75-2.89) (90) | **1.80**  (1.19-2.40) (69) | **2.03**  (1.50-2.556) (89) | **1.93**  (1.32-2.54) (89) | **1.93**  (1.40-2.46) (87) |
| **Central Nervous System drugs** | 3 months Recall Period | **1.16**  (0.86-1.46) (150) | **0.69**  (0.23-1.16) (65) | **1.52**  (1.14-1.89) (85) | **0.52**  (0.23-0.80) (64) | **1.64**  (1.19-2.09) (86) | **0.97**  (0.47-1.47) (65) | **1.31**  (0.94-1.67) (85) |
|  | 1 year retrospective | **4.43**  (3.36-5.49) (162) | **2.53**  (0.90-4.16) (72) | **5.94**  (4.60-7.29) (90) | **1.77**  (0.84-2.71) (71) | **6.49**  (4.80-8.13) (91) | **3.82**  (2.02-5.63) (74) | **4.93**  (3.68-6.19) (88) |
|  | 5 year retrospective | **16.36**  (12.24-20.49) (176) | **21.36**  (16.3-26.4) (80) | **10.36**  (3.73-17.0) (96) | **6.24**  (2.99-9.49) (80) | **24.80**  (18.2-31.5) (96) | **13.21**  (6.56-19.9) (84) | **19.24**  (14.2-24.3) (92) |
|  | 6 months prospective | **2.25**  (1.70-2.80) (158) | **1.41**  (0.52-2.30) (68) | **2.89**  (2.21-3.57) (90) | **0.83**  (0.36-1.29) (69) | **3.36**  (2.51-4.21) (89) | **1.70**  (0.82-2.59) (89) | **2.70**  (2.00-3.40) (87) |
| **Gastro-intestinal system drugs** | 3 months Recall Period | **1.03**  (0.79-1.26) (150) | **0.62**  (0.33-0.90) (65) | **1.34**  (0.99-1.69) (85) | **0.61**  (0.31-0.91) (64) | **1.34**  (1.00-1.68) (86) | **0.68**  (0.38-0.97) (65) | **1.29**  (0.95-1.64) (85) |
|  | 1 year retrospective | **3.80**  (3.00-4.60) (162) | **2.10**  (1.18-3.02) (72) | **5.17**  (3.98-6.35) (90) | **1.92**  (0.98-2.87) (71) | **5.26**  (4.11-6.41) (91) | **2.11**  (1.24-2.98) (74) | **5.23**  (4.00-6.45) (88) |
|  | 5 year retrospective | **14.06**  (10.99-17.14) (176) | **18.99**  (14.4-23.6) (80) | **8.65**  (4.52-11.8) (96) | **7.51**  (3.92-11.1) (80) | **19.52**  (15.0-24.1) (96) | **7.63**  (4.33-10.9) (84) | **19.93**  (15.1-24.7) (92) |
|  | 6 months prospective | **1.92**  (1.50-2.35) (158) | **1.06**  (0.58-1.54) (68) | **2.58**  (1.95-3.21) (90) | **1.06**  (0.58-1.54) (69) | **2.60**  (1.96-3.23) (89) | **0.96**  (0.52-1.40) (89) | **2.71**  (2.06-3.36) (87) |
| **Eye Drugs** | 3 months Recall Period | **0.19**  (0.08-0.30) (150) | **0.06**  (0.00-0.12) (65) | **0.29**  (0.11-0.48) (85) | **0.05**  (0.00-0.10) (64) | **0.30**  (0.12-0.49) (86) | **0.08**  (0.00-0.16) (65) | **0.28**  (0.10-0.47) (85) |
|  | 1 year retrospective | **0.67**  (0.32-1.03) (162) | **0.19**  (0.04-0.35) (72) | **1.06**  (0.43-1.68) (90) | **0.14**  (0.01-0.27) (71) | **1.08**  (0.47-1.71) (91) | **0.15**  (0.02-0.27) (74) | **1.11**  (0.47-1.75) (88) |
|  | 5 year retrospective | **2.22**  (1.00-3.44) (176) | **3.43**  (1.23-5.63) (80) | **0.78**  (0.35-1.20) (96) | **0.46**  (0.20-0.72) (80) | **3.69**  (1.48-5.89) (96) | **0.71**  (0.31-1.12) (84) | **3.60**  (1.31-5.89) (92) |
|  | 6 months prospective | **0.34**  (0.14-0.55) (158) | **0.10**  (0.00-0.21) (68) | **0.52**  (0.17-0.87) (90) | **0.09**  (-0.01-0.19) (69) | **0.54**  (0.19-0.89) (89) | **0.04**  (-0.02-0.10) (89) | **0.59**  (0.22-0.95) (87) |
| **All Other Drugs** | 3 months Recall Period | **1.95**  (1.61-2.29) (150) | **1.65**  (1.22-2.07) (65) | **2.18**  (1.67-2.68) (85) | **1.30**  (0.89-1.71) (64) | **2.43**  (1.94-2.92) (86) | **1.22**  (0.81-1.63) (65) | **2.50**  (2.02-2.99) (85) |
|  | 1 year retrospective | **7.53**  (6.33-8.73) (162) | **5.81**  (4.20-7.41) (72) | **8.91**  (7.19-10.6) (90) | **4.69**  (3.31-6.07) (71) | **9.75**  (8.01-11.5) (91) | **4.24**  (2.85-5.63) (74) | **10.30**  (8.60-12.0) (88) |
|  | 5 year retrospective | **28.60**  (23.68-33.51) (176) | **34.88**  (27.6-42.2) (80) | **21.06**  (15.0-27.2) (96) | **15.03**  (10.1-19.9) (80) | **39.91**  (32.5-47.3) (96) | **14.96**  (9.63-20.3) (84) | **41.04**  (33.8-48.3) (92) |
|  | 6 months prospective | **4.08**  (3.35-4.80) (158) | **3.24**  (2.28-4.19) (68) | **4.71**  (3.67-5.75) (90) | **2.41**  (1.64-3.17) (69) | **5.37**  (4.30-6.44) (89) | **2.31**  (1.52-3.10) (89) | **5.52**  (4.46-6.58) (87) |
