# Supplementary material for: The Cost of Ankylosing Spondylitis in the UK Using Linked Routine and Patient-Reported Survey Data
Source: PLoS One. 2015 Jul 17;10(7):e0126105. doi: 10.1371/journal.pone.0126105 (PMC4506082; doi:10.1371/journal.pone.0126105)
Supplement: S5 Table — (DOCX) [file pone.0126105.s005.docx]

Supplementary Table 5: GP Analysis: Healthcare resource procedures from routine data

| **Procedure** | **Time frames for Routine Data Sets** | **All Patient**  Mean (95% CI) (n) | **BASDAI Group**  Mean (95% CI) (n) | | **BASFI Group**  Mean (95% CI) (n) | | **AGE**  Mean (95% CI) (n) | |
| --- | --- | --- | --- | --- | --- | --- | --- | --- |
|  |  |  | **BASDAI<40** | **BASDAI≥40** | **BASFI<40** | **BASFI≥40** | **Age<50** | **Age≥50** |
| **Average number of Diagnostic Procedures** | 3 months Recall Period | **0.10**  (0.04-0.16) (150) | **0.09**  (0.01-0.18) (65) | **0.11**  (0.02-0.19) (85) | **0.08**  (0.00-0.16) (64) | **0.12**  (0.03-0.20) (86) | **0.06**  (0.00-0.12) (65) | **0.13**  (0.04-0.22) (85) |
|  | 1 year retrospective | **0.35**  (0.24-0.46) (162) | **0.22**  (0.09-0.35) (72) | **0.46**  (0.29-0.62) (90) | **0.23**  (0.09-0.36) (71) | **0.45**  (0.29-0.61) (91) | **0.20**  (0.07-0.34) (74) | **0.48**  (0.31-0.64) (88) |
|  | 5 year retrospective | **1.45**  (1.10-1.81) (176) | **0.96**  (0.58-1.34) (80) | **1.86**  (1.31-2.42) (96) | **0.81**  (0.49-1.13) (80) | **1.99**  (1.42-2.56) (96) | **0.94**  (0.54-1.34) (84) | **1.92**  (1.36-2.48) (92) |
|  | 6 months prospective | **0.18**  (0.11-0.26) (158) | **0.15**  (0.03-0.26) (68) | **0.21**  (0.11-0.31) (90) | **0.13**  (0.02-0.24) (69) | **0.22**  (0.12-0.33) (89) | **0.13**  (0.04-0.22) (71) | **0.23**  (0.11-0.35) (87) |
| **Average number of Laboratory Procedures** | 3 months Recall Period | **0.85**  (0.61-1.08) (150) | **0.69**  (0.41-0.97) (65) | **0.96**  (0.61-1.32) (85) | **0.59**  (0.32-0.87) (64) | **1.03**  (0.68-1.39) (86) | **0.62**  (0.34-0.89) (65) | **1.02**  (0.67-1.38) (85) |
|  | 1 year retrospective | **2.91**  (2.22-3.60) (162) | **2.42**  (1.57-3.22) (72) | **3.30**  (2.25-4.35) (90) | **2.01**  (1.21-2.82) (71) | **3.60**  (2.55-4.65) (91) | **2.05**  (1.21-2.90) (74) | **3.63**  (2.58-4.67) (88) |
|  | 5 year retrospective | **11.76**  (9.76-13.8) (176) | **9.76**  (7.17-12.35) (80) | **13.42**  (10.5-16.4) (96) | **8.8**  (6.12-11.48) (80) | **14.22**  (11.38-17.05) (96) | **8.14**  (5.73-10.56) (84) | **15.05**  (12.07-18.04) (92) |
|  | 6 months prospective | **1.63**  (1.27-1.99) (158) | **1.31**  (0.84-1.78) (68) | **1.88**  (1.35-2.40) (90) | **1.06**  (0.65-1.47) (69) | **2.08**  (1.53-2.63) (89) | **1.01**  (0.59-1.43) (71) | **2.14**  (1.60-2.68) (87) |
| **Average number of Radiology** | 3 months Recall Period | **0.10**  (0.04-0.16) (150) | **0.09**  (0.01-0.18) (65) | **0.11**  (0.03-0.18) (85) | **0.09**  (0.01-0.18) (64) | **0.10**  (0.03-0.18) (86) | **0.08**  (0.00-0.16) (65) | **0.12**  (0.04-0.20) (85) |
|  | 1 year retrospective | **0.31**  (0.22-0.41) (162) | **0.26**  (0.13-0.40) (72) | **0.36**  (0.22-0.49) (90) | **0.23**  (0.10-0.35) (71) | **0.38**  (0.25-0.52) (91) | **0.27**  (0.14-0.40) (74) | **0.35**  (0.22-0.49) (88) |
|  | 5 year retrospective | **1.35**  (1.08-1.63) (176) | **0.98**  (0.07-1.28) (80) | **1.67**  (1.23-2.10) (96) | **0.80**  (0.55-1.05) (80) | **1.81**  (1.37-2.26) (96) | **0.94**  (0.65-1.23) (84) | **1.23**  (1.28-2.18) (92) |
|  | 6 months prospective | **0.14**  (0.08-0.20) (158) | **0.09**  (0.02-0.16) (68) | **0.18**  (0.09-0.26) (90) | **0.07**  (0.01-0.14) (69) | **0.19**  (0.10-0.28) (89) | **0.08**  (0.02-0.15) (71) | **0.18**  (0.09-0.27) (87) |
| **Average number of Preventive Procedure** | 3 months Recall Period | **0.44**  (0.32-0.56) (150) | **0.34**  (0.19-0.49) (65) | **0.52**  (0.33-0.70) (85) | **0.36**  (0.22-0.53) (64) | **0.49**  (0.31-0.67) (86) | **0.28**  (0.09-0.47) (65) | **0.56**  (0.40-0.73) (85) |
|  | 1 year retrospective | **2.11**  (1.66-2.56) (162) | **1.32**  (0.94-1.70) (72) | **2.74**  (2.01-3.48) (90) | **1.42**  (0.98-1.86) (71) | **2.65**  (1.93-3.36) (91) | **1.28**  (0.80-1.77) (74) | **2.81**  (2.11-3.51) (88) |
|  | 5 year retrospective | **7.76**  (6.34-9.17) (176) | **5.31**  (3.90-6.73) (80) | **9.79**  (7.53-12.1) (96) | **4.95**  (3.67-6.23) (80) | **10.09**  (7.80-12.4) (96) | **4.99**  (3.55-6.43) (84) | **10.28**  (8.01-12.56) (92) |
|  | 6 months prospective | **1.53**  (1.20-1.86) (158) | **1.04**  (0.71-1.38) (68) | **1.90**  (1.39-2.41) (90) | **0.84**  (0.53-1.15) (69) | **2.07**  (1.55-2.58) (89) | **0.94**  (0.60-1.29) (71) | **2.01**  (1.50-2.52) (87) |
| **Average number of Operative Procedure** | 3 months Recall Period | **0.11**  (0.05-0.16) (150) | **0.08**  (0.01-0.14) (65) | **0.13**  (0.05-0.21) (85) | **0.06**  (0.00-0.12) (64) | **0.14**  (0.06-0.22) (86) | **0.06**  (0.00-0.12) (65) | **0.14**  (0.06-0.22) (85) |
|  | 1 year retrospective | **0.45**  (0.33-0.57) (162) | **0.40**  (0.24-0.57) (72) | **0.49**  (0.32-0.66) (90) | **0.31**  (0.16-0.46) (71) | **0.56**  (0.39-0.73) (91) | **0.32**  (0.19-0.46) (74) | **0.56**  (0.38-0.74) (88) |
|  | 5 year retrospective | **2.60**  (1.13-4.06) (176) | **1.46**  (1.03-1.90) (80) | **3.54**  (0.88-6.21) (96) | **1.10**  (0.77-1.43) (80) | **3.84**  (1.18-6.51) (96) | **1.25**  (0.88-1.62) (84) | **3.83**  (1.05-6.60) (92) |
|  | 6 months prospective | **0.34**  (0.22-0.46) (158) | **0.24**  (0.10-0.37) (68) | **0.41**  (0.23-0.59) (90) | **0.17**  (0.06-0.29) (69) | **0.46**  (0.27-0.65) (89) | **0.28**  (0.10-0.46) (71) | **0.38**  (0.22-0.54) (87) |
| **Average number of Other Therapeutic Procedure** | 3 months Recall Period | **0.53**  (0.40-0.66) (150) | **0.32**  (0.70-0.44) (65) | **0.68**  (0.48-0.89) (85) | **0.30**  (0.17-0.43) (64) | **0.70**  (0.50-0.90) (86) | **0.35**  (0.20-0.51) (65) | **0.66**  (0.46-0.85) (85) |
|  | 1 year retrospective | **2.48**  (2.07-2.89) (162) | **1.61**  (1.25-1.97) (72) | **3.18**  (2.53-3.83) (90) | **1.44**  (1.14-1.74) (71) | **3.30**  (2.65-3.95) (91) | **2.04**  (1.46-2.62) (74) | **2.85**  (2.28-3.43) (88) |
|  | 5 year retrospective | **10.11**  (8.68-11.53) (176) | **7.90**  (5.72-10.08) (80) | **11.95**  (10.1-13.8) (96) | **6.0**  (4.96-7.04) (80) | **13.53**  (11.27-15.79) (96) | **7.07**  (5.63-8.52) (84) | **12.88**  (10.62-15.14) (92) |
|  | 6 months prospective | **1.36**  (1.10-1.62) (158) | **0.96**  (0.64-1.27) (68) | **1.67**  (1.29-2.04) (90) | **0.80**  (0.54-1.05) (69) | **1.80**  (1.41-2.19) (89) | **1.08**  (0.76-1.41) (71) | **1.59**  (1.20-1.97) (87) |
