# Supplementary material for: The Cost of Ankylosing Spondylitis in the UK Using Linked Routine and Patient-Reported Survey Data
Source: PLoS One. 2015 Jul 17;10(7):e0126105. doi: 10.1371/journal.pone.0126105 (PMC4506082; doi:10.1371/journal.pone.0126105)
Supplement: S6 Table — (DOCX) [file pone.0126105.s006.docx]

Supplementary Table 6: Distribution of healthcare cost stratified by disease severity, functional ability and age

| **Cost Items** | **All Patient**  Mean (95% CI) (n=400) | | **BASDAI Group**  Mean (95% CI) (n) | | | | | **BASFI Group**  Mean (95% CI) (n) | | **AGE**  Mean (95% CI) (n) | | |
| --- | --- | --- | --- | --- | --- | --- | --- | --- | --- | --- | --- | --- |
|  |  |  | **BASDAI<40**  (n=188) | | | **BASDAI≥40**  (n=212) | | **BASFI<40**  (n=175) | **BASFI≥40**  (n=225) | **Age<50**  (n=150) | **Age≥50**  (n=250) | |
| **Panel A: Distribution of costs among the various types of consultants (at the outpatient set-up)** | | | | | | | | | | | | |
| Rheumatologist | **267**  (232-302) | | | **180**  (140-220) | | **344**  (290-398) | | **187**  (142-232) | **329**  (278-379) | **334**  (273-396) | **226**  (184-268) | |
| Physiotherapist | **174**  (124-225) | | | **79**  (40-118) | | **259**  (171-347) | | **97**  (48-145) | **235**  (153-316) | **148**  (66-230) | **190**  (125-255) | |
| Radiology | **66**  (46-86) | | | **53**  (26-80) | | **77**  (49-106) | | **42**  (21-63) | **85**  (54-116) | **67**  (35-99) | **66**  (41-91) | |
| Nurse | **78**  (59-97) | | | **61**  (33-88) | | **94**  (67-120) | | **60**  (33-87) | **92**  (65-119) | **74**  (43-104) | **81**  (56-106) | |
| Other specialist | **170**  (122-217) | | | **130**  (78-182) | | **205**  (128-281) | | **120**  (67-173) | **209**  (136-282) | **118**  (64-171) | **201**  (133-270) | |
| Total cost of Visits to Consultants/Professional | **755**  (654-856) | | | **503**  (392-614) | | **978**  (821-1136) | | **505**  (386-624) | **949**  (800-1098) | **740**  (591-890) | **764**  (629-898) | |
| **Panel B: Distribution of costs among various types of Tests and Imaging for the AS patients** | | | | | | | | | | | | |
| Bone scan | **17**  (12-22) | | | | **11**  (6-16) | | **23**  (15-30) | **12**  (6-17) | **22**  (14-29) | **14**  (8-20) | | **20**  (13-26) |
| X-ray/MRI/CT scan | **77**  (62-91) | | | | **45**  (27-63) | | **104**  (82-127) | **45**  (26-64) | **101**  (80-122) | **81**  (61-101) | | **74**  (53-94) |
| Pathology (Blood/Urine) | **25**  (21-29) | | | | **19**  (14-25) | | **29**  (24-35) | **19**  (14-25) | **29**  (23-35) | **27**  (20-34) | | **23**  (18-28) |
| Other tests (incl. Gastroscopy) | **12**  (7-17) | | | | **9**  (2-16) | | **14**  (7-21) | **6**  (0.5-12) | **16**  (9-24) | **12**  (3-20) | | **12**  (6-18) |
| Total cost | **130**  (110-150) | | | | **85**  (61-108) | | **171**  (140-201) | **82**  (59-105) | **168**  (138-197) | **133**  (107-159) | | **129**  (100-157) |
| **Panel C: Distribution of costs among various types of Therapies for the AS patients** | | | | | | | | | | | | |
| **NHS therapies** | | | | | | | | | | | | |
| NHS Physiotherapy | | **68**  (57-78) | | | **49**  (35-62) | | **85**  (70-100) | **60**  (45-75) | **75**  (60-88) | **78**  (60-96) | **62**  (49-75) | |
| NHS Hydrotherapies | | **39**  (31-48) | | | **27**  (17-38) | | **50**  (37-63) | **29**  (18-41) | **47**  (34-59) | **37**  (24-51) | **40**  (29-51) | |
| NHS Other Therapy | | **54**  (41-67) | | | **30**  (16-45) | | **74**  (53-95) | **30**  (17-44) | **72**  (51-92) | **46**  (26-65) | **58**  (41-76) | |
| **Total NHS Therapy** | | **161**  (138-184) | | | **106**  (80-132) | | **209**  (173-245) | **120**  (93-147) | **193**  (158-227) | **161**  (127-196) | **160**  (130-191) | |
| **Non-NHS therapies** | | | | | | | | | | | | |
| Non-NHS Physiotherapy | | **24**  (17-31) | | | **22**  (12-32) | | **25**  (16-35) | **27**  (16-38) | **22**  (13-31) | **25**  (13-37) | **23**  (15-32) | |
| Non-NHS Hydrotherapies | | **13**  (8-18) | | | **15**  (7-23) | | **11**  (4-18) | **11**  (3-18) | **15**  (7-22) | **11**  (3-19) | **14**  (7-21) | |
| Non-NHS Other Therapy | | **24**  (17-32) | | | **20**  (10-31) | | **28**  (17-39) | **21**  (10-32) | **26**  (16-37) | **28**  (14-42) | **22**  (13-31) | |
| **Total Non-NHS Therapy** | | **61**  (48-74) | | | **58**  (38-77) | | **64**  (46-82) | **59**  (40-78) | **63**  (45-81) | **64**  (42-86) | **59**  (43-76) | |
