# Supplementary material for: The Cost of Ankylosing Spondylitis in the UK Using Linked Routine and Patient-Reported Survey Data
Source: PLoS One. 2015 Jul 17;10(7):e0126105. doi: 10.1371/journal.pone.0126105 (PMC4506082; doi:10.1371/journal.pone.0126105)
Supplement: S7 Table — (DOCX) [file pone.0126105.s007.docx]

Supplementary Table 7: Out of Pocket AS-related costs for the AS Patients (£/AS patient/year)

| **Cost Items** | **All Patient**  Mean (95% CI)  (n = 400) | **BASDAI Group**  Mean (95% CI) (n) | | **BASFI Group**  Mean (95% CI) (n) | | **AGE**  Mean (95% CI) (n) | |
| --- | --- | --- | --- | --- | --- | --- | --- |
|  |  | **BASDAI<40**  (n=188) | **BASDAI≥40**  (n=212) | **BASFI<40**  (n=175) | **BASFI≥40**  (n=225) | **Age<50**  (n=150) | **Age≥50**  (n=250) |
| Adjustment Costs | **192**  (109-275) | **80**  (38-123) | **291**  (139-442) | **48**  (23-73) | **304**  (158-449) | **168**  (-19-355) | **206**  (134-278) |
| Appliances | **7.1**  (3.8-10.3) | **2.14**  (0.44-3.84) | **11.4**  (5.5-17.4) | **1.32**  (0.02-2.62) | **11.5**  (5.9-17.2) | **3.03**  (1.1-5.0) | **9.5**  (4.4-14.6) |
| Exercises | **113**  (85-140) | **93**  (69-117) | **130**  (82-178) | **114**  (81-147) | **112**  (69-154) | **174**  (109-238) | **76**  (55-97) |
| Other costs | **67**  (37-97) | **29**  (12-46) | **101**  (46-156) | **28**  (12-45) | **97**  (45-150) | **30**  (11-48) | **90**  (43-136) |
| Society Costs | **18**  (13-23) | **14**  (7-20) | **22**  (14-29) | **14**  (8-21) | **21**  (13-28) | **15**  (7-24) | **19**  (12-26) |
| On-going Cost | **199**  (37-361) | **49**  (-4-103) | **331**  (30-632) | **36**  (-16-88) | **325**  (41-609) | **22**  (-11-54) | **305**  (47-562) |
| Transport Costs | **58**  (42-74) | **36**  (24-48) | **78**  (49-106) | **24**  (16-31) | **84**  (57-113) | **42**  (29-56) | **67**  (42-92) |
| Over the Counter Medicine | **51**  (44-58) | **40**  (31-49) | **60**  (50-71) | **50**  (39-61) | **52**  (42-62) | **47**  (36-58) | **54**  (44-64) |
| **Total Out of Pocket Expense** | **705**  (498-912) | **343**  (261-425) | **1024**  (646-1402) | **315**  (237-393) | **1007**  (649-1365) | **501**  (286-716) | **826**  (523-1130) |
| One off Purchase Expense | **362**  (110-614) | **149**  (-62-360) | **551**  (114-988) | **155**  (-72-381) | **523**  (111-936) | **173**  (-62-408) | **476**  (97-854) |
