# Supplementary material for: The Cost of Ankylosing Spondylitis in the UK Using Linked Routine and Patient-Reported Survey Data
Source: PLoS One. 2015 Jul 17;10(7):e0126105. doi: 10.1371/journal.pone.0126105 (PMC4506082; doi:10.1371/journal.pone.0126105)
Supplement: S8 Table — (DOCX) [file pone.0126105.s008.docx]

Supplementary Table 8: Impact of AS on on-the-job performance due to physical and emotional problems as assessed by the Work Limitations Questionnaire

| ***How difficult it is to (items 1-19 below)….*** | Percentage of patients responded on an integer scale of 1 (most difficult) to 5 (Least Difficult) | | | | | Mean Score on the response scale 1-5 | Confidence Interval for the score | Number of obs. |
| --- | --- | --- | --- | --- | --- | --- | --- | --- |
|  | (1)  Difficult all of the time (100%) | (2)  Difficult most of the time | (3)  Difficult some of the time (50%) | (4)  Difficult a slight bit of the time | (5)  Difficult none of the time (0%) |  |  |  |
| 1. Work the required number of hours | 37.80 | 36.84 | 13.88 | 9.09 | 2.39 | 1.98 | 1.83-2.12 | 213 |
| 2. Get going easily at the beginning of workday | 24.17 | 35.07 | 20.38 | 14.22 | 6.16 | 2.41 | 2.25-2.57 | 213 |
| 3. Start on job as soon as you arrived at work | 52.20 | 24.88 | 11.71 | 7.32 | 3.90 | 1.78 | 1.64-1.95 | 212 |
| 4. Work without stopping to take breaks or rests | 30.54 | 33.00 | 16.26 | 15.27 | 4.93 | 2.20 | 2.03-2.37 | 213 |
| 5. Stick to a routine or schedule | 45.92 | 29.59 | 11.73 | 10.71 | 2.04 | 1.80 | 1.65-1.96 | 210 |
| 6. Keep your mind on your work | 41.51 | 32.55 | 18.87 | 5.66 | 1.42 | 1.92 | 1.79-2.05 | 213 |
| 7. Think clearly when working | 49.06 | 30.66 | 13.68 | 5.66 | 0.94 | 1.78 | 1.65-1.91 | 213 |
| 8. Do work carefully | 55.71 | 28.57 | 10.48 | 4.29 | 0.95 | 1.64 | 1.52-1.76 | 213 |
| 9. Concentrate on your work | 42.45 | 36.32 | 16.98 | 3.30 | 0.94 | 1.83 | 1.71-1.95 | 213 |
| 10. Work without losing train of thought | 4.57 | 37.26 | 16.98 | 4.25 | 0.94 | 1.87 | 1.75-1.99 | 213 |
| 11. Easily read or use your eyes when working | 51.89 | 29.25 | 11.32 | 5.66 | 1.89 | 1.76 | 1.62-1.89 | 213 |
| 12. Speak in-person, meeting, on phone | 74.51 | 15.69 | 6.37 | 1.96 | 1.47 | 1.34 | 1.23-1.46 | 213 |
| 13. Control temper around people when working | 55.92 | 29.86 | 9.48 | 3.79 | 0.95 | 1.62 | 1.50-1.74 | 213 |
| 14. Help other people to get work done | 63.05 | 21.67 | 10.84 | 3.45 | 0.99 | 1.50 | 1.38-1.63 | 213 |
| 15. Handle the workload | 42.86 | 30.95 | 15.71 | 7.14 | 3.33 | 1.97 | 1.82-2.12 | 210 |
| 16. Work fast enough | 42.72 | 31.07 | 14.08 | 6.80 | 5.34 | 1.96 | 1.80-2.12 | 211 |
| 17. Finish work on time | 47.24 | 31.66 | 11.56 | 4.52 | 5.03 | 1.79 | 1.64-1.95 | 209 |
| 18. Do your work without making mistakes | 54.33 | 30.77 | 10.58 | 2.40 | 1.92 | 1.64 | 1.52-1.77 | 211 |
| 19. Feel you’ve done what you are capable of doing | 48.08 | 23.56 | 15.38 | 7.21 | 5.77 | 1.97 | 1.81-2.14 | 210 |

Note: The values indicate the percent of respondents. Therefore the row total for corresponding questions is 100. Some row totals may not sum up to 100, because of the rounding of the percentages. The following question was asked: “ In the past 2 weeks, how much of the time did your physical health or emotional problems make it difficult for you to do the following?”.
