# Supplementary material for: The Cost of Ankylosing Spondylitis in the UK Using Linked Routine and Patient-Reported Survey Data
Source: PLoS One. 2015 Jul 17;10(7):e0126105. doi: 10.1371/journal.pone.0126105 (PMC4506082; doi:10.1371/journal.pone.0126105)
Supplement: S9 Table — (DOCX) [file pone.0126105.s009.docx]

Supplementary Table 9: Determinants of AS related work productivity loss costs

|  | **Logit Model** | | **Multivariate Regression Model** | | | | | |
| --- | --- | --- | --- | --- | --- | --- | --- | --- |
|  | **Dependent Variables** | | | | | | | |
|  | Retirement Reason  (n = 216) | Require unpaid assistance  (n = 413) | Productivity Loss in Daily regular activity for people at work  (n = 219) | Productivity Loss at work  (n=219) | Average difficulty  (n=223) | Average ability  (n=223) | Cost absenteeism  (n=206) | Cost presenteeism  (n=206) |
| **Male Gender** | **-0.44**  (-1.32, -0.43) | **-0.67****  (-1.21: -0.12) | **-0.78****  (-1.40:-0.17) | **-0.29**  (-0.87, 0.29) | **0.10**  (-0.08, 0.29) | **0.074**  (-0.22-0.36) | **-532**  (-1628, 563) | **1530**  (-630, 3690) |
| **Age** | **-0.03***  (-0.6, -0.00) | **0.001**  (-0.02-0.02) | -**0.022***  (-0.05-0.00) | **-0.023***  ( -0.0.5, 0.00) | **-0.01****  (-0.02, 0.00) | **-0.00**  (CI -0.01, 0.01) | **-49.4****  (-96.5, -2.3) | **-49**  (-141, 44) |
| **BASFI** | **0.015****  (0.00, 0.03) | **0.032****  (0.02-0.04) | **0.033****  (0.02-0.05) | **0.034****  ( 0.02, 0.05) | **0.007****  (0.002, 0.010) | **-0.006***  (CI -0.013, 0.00) | **48****  (20.4, 75.7) | **92.7****  (38, 147) |
| **EQ5D** | **-0.74**  (-2.03, 0.55) | **-1.06****  ( -1.98, - 0.15) | **-4.26****  (-5.66, 2.79) | **-2.17****  (-3.64, 0.93) | **-1.07****  ( -1.51, 0.63) | **0.62***  (CI -0.062, 1.29) | **-904**  (-3680, 1871) | **-10486****  (-15959, 5012) |
| **Correctly Classified** | **75.0%** | **74.6%** | **VIF: 1.41** | **VIF: 1.41** | **VIF: 1.40** | **VIF: 1.40** | **VIF: 1.48** | **VIF: 1.48** |

Note: VIF: Variance Inflation Factor. ** and * denote coefficients significant at 1% level and 5% level significance. 95% CI are reported in the parentheses.
