# Supplementary material for: The Cost of Ankylosing Spondylitis in the UK Using Linked Routine and Patient-Reported Survey Data
Source: PLoS One. 2015 Jul 17;10(7):e0126105. doi: 10.1371/journal.pone.0126105 (PMC4506082; doi:10.1371/journal.pone.0126105)
Supplement: S11 Table — (DOCX) [file pone.0126105.s011.docx]

Supplementary Table 11: Cost estimates of unpaid assistance

| **Cost Components** | **All patients**  **(n = 437)** | **BASDAI Group**  **Mean (95% CI) (n)** | | **BASFI Group**  **Mean (95% CI) (n)** | | **Age** | |
| --- | --- | --- | --- | --- | --- | --- | --- |
|  |  | **BASDAI<40**  **(n = 193)** | **BASDAI≥40**  **(n = 244)** | **BASFI<40**  **(n = 185)** | **BASFI≥40**  **(n = 252)** | **Age<50**  **(n = 165)** | **Age≥50**  **(n = 272)** |
| **Help from any care-giver**  **Mean hours/3months** | **51.9**  (CI: 36.8-67.0) | **21.6**  (CI: 6.7-36.6) | **75.8**  (CI: 51.8-99.8) | **9.4**  **(**CI: 1.0-17.9) | **83.0**  (CI: 58-108) | **30.4**  (CI: 11.8-48.9) | **64.9**  (CI: 43-86) |
| **Cost of unpaid assistance (lower bound estimate at minimum wage)**  (Cost(£)/year/patients) | **1279**  (CI: 903-1655) | **538**  (CI: 163-912) | **1865**  (CI: 1268-2463) | **232**  (CI: 21-443) | **2048**  (CI: 1429-2666) | **752**  (CI: 288-1216) | **1599**  (CI: 1065-2132) |
| **Cost of unpaid assistance at mean wage**  (Cost(£)/year/patients) | **2983**  (CI: 2105-3860) | **1254**  (CI: 380-2128) | **4350**  (CI: 2957-5743) | **541**  (CI: 48-1033) | **4776**  (CI: 3333-6218) | **1754**  (CI: 671-2836) | **3728**  (CI: 2484-4973) |
| **Cost of unpaid assistance for health care visits at mean wage**  (Cost(£)/year/patients) | **193**  (CI: 91-295) | **56**  (CI: 26-85) | **302**  (CI: 121-483) | **51**  (CI: 26-75) | **298**  (CI: 122-474) | **137**  **(**CI: 62-213) | **227**  (CI: 69-385) |
| *GP visits* | *42*  *(CI: 23-61)* | *15*  *(CI: -2-31)* | *64*  *(CI: 32-95)* | *8*  *(CI: -2-17)* | *67*  *(CI: 35-100)* | *16*  *(CI: -3-34)* | *58*  *(CI: 29-87)* |
| *Outpatient visits* | *133*  *(CI: 44-223)* | *30*  *(CI: 11-50)* | *214*  *(CI: 55-374)* | *29*  *(CI: 11-47)* | *209*  *(CI: 55-364)* | *116*  *(CI: 54-178)* | *143*  *(CI: 4-283)* |
| *Inpatient visits* | *10*  *(CI: 4-16)* | *6*  *(CI: -1-13)* | *13*  *(CI: 4-13)* | *8*  *(CI: -1-16)* | *12*  *(CI: 3-21)* | *6*  *(CI: -1-12)* | *13*  *(CI: 4-22)* |
| *A & E visits* | *8*  *(CI: 2-14)* | *5*  *(CI: -2-12)* | *11*  *(CI: 2-19)* | *6*  *(CI: -1-14)* | *9*  *(CI: 1-17)* | *0*  *(CI: 0-0)* | *13*  *(CI: 4-22)* |
